# Targeted ultra-deep sequencing unveils a lack of driver-gene mutations linking non-hereditary gastrointestinal stromal tumors and highly prevalent second primary malignancies: random or nonrandom, that is the question

**DOI:** 10.18632/oncotarget.12452

**Published:** 2016-10-28

**Authors:** Bo-Ru Lai, Yu-Tung Wu, Yung-Chia Kuo, Hung-Chih Hsu, Jen-Shi Chen, Tse-Ching Chen, Ren-Chin Wu, Cheng-Tang Chiu, Chun-Nan Yeh, Ta-Sen Yeh

**Affiliations:** ^1^ Department of Surgery, Chang Gung Memorial Hospital at Linkou, Chang Gung University College of Medicine, Taoyuan, Taiwan; ^2^ Department of Hematology-Oncology, Chang Gung Memorial Hospital at Linkou, Chang Gung University College of Medicine, Taoyuan, Taiwan; ^3^ Department of Pathology, Chang Gung Memorial Hospital at Linkou, Chang Gung University College of Medicine, Taoyuan, Taiwan; ^4^ Department of Gastroenterology, Chang Gung Memorial Hospital at Linkou, Chang Gung University College of Medicine, Taoyuan, Taiwan

**Keywords:** GIST, second primary cancer, cancer driver gene, c kit, colorectal cancer

## Abstract

The association of non-hereditary (sporadic) gastrointestinal stromal tumors (GISTs) and second primary malignancies is known to be nonrandom, although the underlying molecular mechanisms remain unknown. In this study, 136 of 749 (18.1%) patients with sporadic GISTs were found to have additional associated cancers, with gastrointestinal and genitourinary/gynecologic/breast cancers being the most prevalent. Gene mutations in GISTs and their associated colorectal cancers (CRCs) (n=9) were analyzed using a panel of 409 cancer-related genes, while a separate group of 40 sporadic CRCs not associated with GISTs served as controls. All 9 of the GISTs had either *KIT* (8 of 9) or *PDGFRA* (1 of 9) mutations that were not present in their associated CRCs. Conversely, all but one of the 9 GIST-associated CRCs exhibited an *APC* mutation, a *TP53* mutation or both, while none of their corresponding GISTs harbored either *APC* or *TP53* mutations. The genetic profile of CRCs with and without associated GISTs did not differ. Although population-based studies and case series worldwide, including ours, have unanimously indicated that the GIST-CRC association is nonrandom, our targeted ultra-deep sequencing unveiled a lack of driver-gene mutations linking sporadic GISTs to highly prevalent second primaries. Further studies are needed to elucidate other genetic alterations that may be responsible for this puzzling contradiction.

## INTRODUCTION

Gastrointestinal stromal tumors (GISTs) are the most common mesenchymal neoplasm of the gastrointestinal (GI) tract, with an incidence of between 7 and 20 cases per million. The most common locations are the stomach (60 to 70%) and the small bowel (25 to 30%), with tumors of the large bowel (5%) and the omentum/mesentery (5%) being less common [1]. A key event in the carcinogenesis of GIST is the acquisition of gain-of-function mutations in the genes encoding the receptor tyrosine kinase *KIT* (80-90%) or platelet derived growth factor receptor α (*PDGFRA*) (5-10%) [1]. Hereditary GIST syndromes are caused by germline genomic alterations in the *KIT*, *PDGFRA*, neurofibromin-1, and succinate dehydrogenase genes, which are phenotypically associated with multiple additional benign and malignant neoplasms [2, 3]. On the other hand, non-hereditary (sporadic) GISTs are associated with second malignancies at rates of between 14 and 25% [4–10]. The mechanisms by which sporadic GISTs are coupled to synchronous or metachronous malignancies remain elusive. To address this important gap in the literature, we analyzed the prevalence and patterns of GISTs associated with second malignancies in patients at our institute, and for the first time comprehensively surveyed the somatic gene mutations in GISTs and their corresponding second malignancies, using a panel of 409 cancer-related genes.

## RESULTS

**Figure 1:**
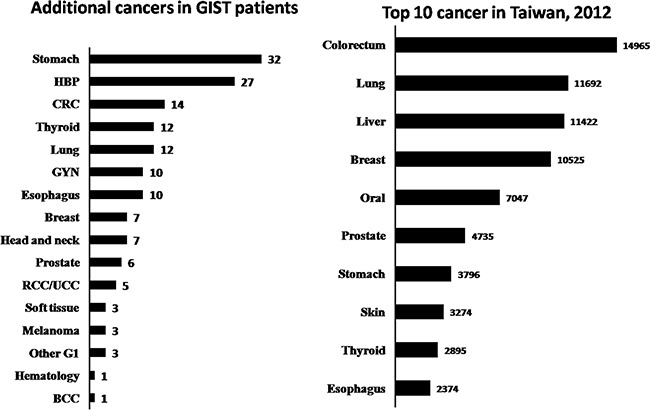
Comparison of ranks of the additional malignancies in GIST patients, in decreasing order, with the top-10 cancers that occurred in Taiwan in 2012

**Figure 2:**
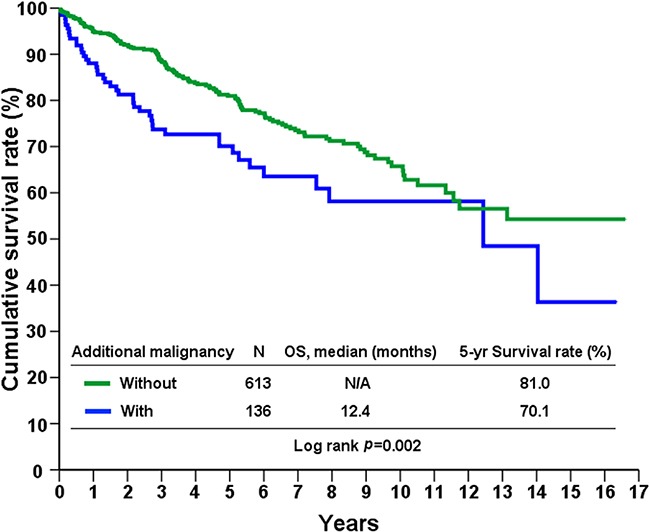
Overall survival of GIST patients with and without second primary malignancies

A total of 749 patients with sporadic GISTs were identified at our institute between 1995 and 2015, of which 136 (18.1%) had additional cancers. Of these 136 patients, 115 had one, 19 had two, and 2 had three additional cancers. The patients were categorized into three groups, based on the timing of GIST occurrence with respect to their additional cancers, as follows: in group 1, the GIST occurred before other cancers (n=22); in group 2, the GIST occurred after other cancers (n=50), and in group 3, the GIST and additional cancers occurred synchronously (n=64). From group 1, 13 of the 22 patients (59%) received either adjuvant or palliative imatinib mesylate treatment after GIST-related surgery. The demographic and clinicopathologic data of 131 of the GIST patients are presented in Table 1. Group 1 patients were found, on average, to be younger, with larger tumors, cancers with higher mitotic rates, and thus higher risk potential. The anatomic distribution of additional cancers among the three groups is shown in Table 2. GI and genitourinary (GU)/gynecologic (GYN)/breast cancers were found to be the two most prevalent groups of associated malignancies based on anatomic distribution. The prevalence ranking, in decreasing order, of the GIST-associated cancers was compared with that of the top-10 most prevalent cancers in Taiwan in 2012, and a remarkable disaccord was observed (Figure 1). The 5-year overall survival of GIST patients with additional associated cancers was inferior to that of patients with GIST alone (69.1% vs. 80.9%, p<0.001, log-rank test) (Figure 2).

**Table 1:** Demographics and clinicopathologic data of GIST associated with additional cancers

Characteristics of GIST	GIST before cancer	GIST after cancer	Synchronous	Total	p-value
	n=22	n=50	n=64	n=136	
Male	11 (50.0%)	22 (44.0%)	42 (65.6%)	75 (55.1%)	0.064
Age at GIST (years)					<0.001
Median (range)	54.5 (41-81)	70.0 (48-89)	69.2 (29-87)	68.2 (29-89)	
Anatomic site					0.068
Stomach	10 (45.5%)	32 (64.0%)	47 (73.4%)	89 (65.4%)	
Small bowel	8 (36.4%)	14 (28.0%)	14 (21.9%)	36 (26.5%)	
Colo-rectum	2 (9.1%)	1 (2.0%)	3 (4.7%)	6 (4.4%)	
E-GIST	2 (9.1%)	3 (6.0%)	0	5 (3.7%)	
Mitotic rate (HPF)					0.023
<5/50	10 (45.5%)	33 (66.0%)	48 (75.0%)	91 (66.9%)	
>5/50	9 (40.9%)	13 (26.0%)	7 (10.9%)	29 (21.3%)	
Size of tumor (cm)					0.001
Median (range)	8.5 (1.8-20.5)	4.4 (0.4-20.1)	2.5 (0.2-28.0)	4.0 (0.2-28.0)	
Risk potential*					0.001
Very low/low	6 (30.0%)	22 (47.8%)	41 (70.7%)	69 (55.6%)	
Intermediate	1 (5.0%)	9 (19.6%)	8 (13.8%)	18 (14.5%)	
High	13 (65.0%)	15 (32.6%)	9 (15.5%)	37 (29.8%)	

GIST, gastrointestinal stromal tumor; E-GIST, extra-gastrointestinal tract GIST.

* not all data available.

**Table 2:** Anatomic distribution of additional cancers in patients with GIST

Site of additional cancers	GIST before cancer	GIST after cancer	Synchronous	Total	P value
No. of cases	25	63	71	160	
GI tract	8 (32.0)	24 (38.1%)	59 (83.1%)	91 (57.2%)	0.001
Stomach	2	5	24	31	
Colorectal	2	8	10	20	
Esophagus	0	3	7	10	
HPB	4	8	15	27	
Other GI	0	0	3	3	
GU/GYN/Breast	3 (12.0%)	19 (30.2%)	7 (10.6%)	29 (18.8%)	
Prostate	0	6	0	6	
RCC/UCC	1	2	3	6	
GYN/Genital	1	6	3	10	
Breast	1	5	1	7	
Lung	6 (24.0%)	3 (4.8%)	4 (6.1%)	13 (8.4%)	
Skin	1 (4.0%)	2 (3.2%)	0	4 (2.6%)	
Melanoma	1	1	0	2	
BCC	0	1	0	1	
Hematology	0	1 (1.6%)	0	1 (0.6%)	
Others	7 (28.0%)	14 (22.2%)	1 (1.5%)	22 (14.3%)	
Thyroid	5	5	0	10	
Sarcoma	1	2	0	3	
Head and neck	1	7	1	9	

GI, gastrointestinal; HPB, hepatopancreaticobiliary; GU, genitourinary; GYN, gynecologic; RCC, renal cell carcinoma; UCC, urothelial cell carcinoma; BCC, basal cell carcinoma.

To investigate the randomness or otherwise of the association between GISTs and their additional cancers, we performed massively parallel next-generation sequencing, of a panel of 409 cancer-related genes, on the 9 GIST-CRC tumor pairs. In addition, we recruited another independent cohort of patients with sporadic metastatic CRCs without GIST (n=40) to serve as controls, with the aim of detecting any aberrant gene mutations in associated GIST-CRC pairs. Clinical descriptions of the 9 patients with paired GISTs and CRCs, as well as the sites and sizes of their tumors, are shown in Table 3. All samples were sequenced at an average depth of >1000x (Supplementary Table S2). Raw sequence reads were aligned to the hg19 human reference genome in order to identify variants. Following annotation, variants were filtered to remove single nucleotide polymorphisms (SNPs) and synonymous mutations. Only non-synonymous mutations were used in subsequent analyses.

**Table 3:** Clinical information of GISTs associated with colorectal cancers

Code of patients	Age	Gender	GIST site	GIST size (cm)	CRC site	CRC size (cm)
1	73	M	Stomach	4.5	Rectum	6.0
2	74	M	Jejunum	4.0	Rectum	3.4
3	60	M	Stomach	7.0	D-colon*	5.0
4	74	M	Jejunum	2.0	Sigmoid	4.5
5	87	M	Jejunum	7.5	Rectum	5.0
6	80	F	Jejunum	11.5	A-colon	5.6
7	66	M	Jejunum	2.0	Rectum	2.2
8	67	F	Stomach	1.7	Rectum*	4.5
9	76	F	Stomach	2.4	T-colon*	3.8

* GIST after cancer; while the remaining 6 patients had their GISTs and colorectal cancers identified synchronously.

GIST, gastrointestinal stromal tumor; CRC, colorectal cancer.

**Figure 3:**
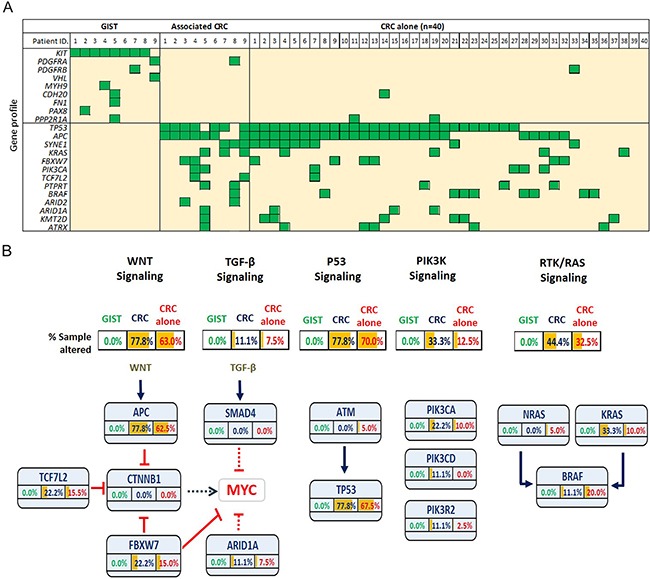
**A.** Summary of the gene mutations in GISTs (n=9) and associated colorectal cancers (CRCs), with an independent cohort of sporadic CRCs without GIST (n=40) serving as a control. **B.** Proportion of mutated individual genes, involved in various signaling pathways, in GISTs and in CRCs with and without associated GISTs.

We detected 164 non-synonymous mutations in 109 genes, within the 9 sample pairs (Supplementary Table S3). All GISTs harbored activating mutations in either *KIT* (n=8, 88.9%) or *PDGFRA* (n=1, 11.1%), however no corresponding mutations were detected in the paired CRC tumors (Figure 3A). Consistent with previous observations, all of the *KIT* mutations were located within hotspot exons, with the most prevalent hotspot, hosting 6 of the 8 alterations, found within exon 11, a region that encodes the juxtamembrane domain of the kinase (Supplementary Figure S1). Other genes with a single unshared mutation that were identified in GISTs include *PDGFRB, MYH9, PAX8, PDGFRB, CDH20, FN1*, and *PPP2R1A*. In the paired CRCs, the most prevalently mutated genes were *APC* (n=7, 77.8%), *TP53* (n=7, 77.8%), *SYNE1* (n=3, 33.3%), *KRAS* (n=3, 33.3%), *FKBW7* (n=2, 22.2%), and *PIK3CA* (n=2, 22.2%), all of which are commonly altered in CRCs (Figure 3A). Again, none of these mutations were detected in the paired GIST. To further describe the individual genes involved in CRCs with and without GIST, we delineated the mutation events into five main oncogenic signaling pathways (Figure 3B). We found that identical pathways were involved in CRCs with and without GIST.

## DISCUSSION

In the present study, we report one of the world's largest surgical series of sporadic GISTs associated with second primary malignancies, treated at a single institute [5, 7, 8, 10]. After excluding benign neoplasms and hereditary GISTs, the occurrence rate of GISTs associated with second malignancies was 18.1% (136 of 749 patients), with GI tract and GU/GYN/breast cancers being most common. Importantly, although the overall survival of patients in the three groups described above was not statistically significantly different (data not shown), the 5-year survival of patients with GISTs associated with additional malignancies was significantly inferior to those with GIST alone (69.1% vs 80.9%, p<0.001).

The reason for the high percentage of additional malignancies that accompany GIST is not yet clear. Although there is no direct evidence to support the theory that the treatment of leukemia with imatinib increases the risk of additional malignancies [11, 12], Kanda et al. found that GIST patients in the imatinib era tended to have a higher incidence of additional malignancies than those from the pre-imatinib era [13]. Some might argue that an underlying genetic instability or mismatch repair defect could lead to the mutation of *KIT*, resulting in GIST, while also activating oncogenes and/or inactivating tumor suppressor genes to promote additional malignancies. Accordingly, we compared the gene mutations in GISTs and their corresponding second cancers, using a genomic panel that comprehensively encompasses 12 carcinogenic signaling pathways. Pathways involved in DNA repair, the cell cycle, and chromosome stability, as well as RAS, PI3K, MAPK, TGF-β, NOTCH, Wnt, and Hedgehog signaling, were investigated, as were transcription factors and chromatin remodeling genes [14]. This comparison enabled us to identify which, if any, gene mutations were shared by GISTs and associated malignancies, in order to explain their genetic link.

Our understanding of the molecular events underlying CRC is far greater than that of other solid malignancies, which helps to narrow the scope of potential genetic links [15–18]. For example, germline mutations, such as in the *APC* gene, are responsible for the inherited form of CRC, while a stepwise accumulation of somatic mutations, in genes such as *APC*, *KRAS*, and *TP53*, is thought to underline most sporadic CRCs [17]. Mismatch repair genes, including *MSH2*, *MLH1*, *PMS1*, *PMS2*, *MSH6*, and *MLH3*, are responsible for correcting the frequent nucleotide mis-pairings and insertions or deletions that occur during DNA replication [19]. All of genes above, recognized in sporadic CRC development, were encompassed in our targeted gene panel.

Disappointingly, but not unexpectedly, our genetic study failed to yield any genetic link between GISTs and their corresponding CRCs. All 9 GISTs exhibited either a *KIT* or a *PDGFRA* mutation exclusively. One of the associated CRCs displayed a *PDGFRA* mutation; however, in this patient, the corresponding GIST exhibited a *KIT* mutation rather than a *PDGFRA* mutation. Similarly, 8 of the 9 GIST-associated CRCs harbored an *APC* mutation, a *TP53* mutation or both, while none of their corresponding GISTs exhibited either an *APC* or a *TP53* mutation. When we investigated the involvement of five oncogenic pathways, including Wnt, TGF-β, p53, PI3K, and RTK/Ras signaling pathways, there was clearly no distinct difference between the CRCs with and without GIST. Most importantly, none of the GISTs with associated CRCs were genetically involved in these pathways. Taken together, we might speculate that the association between GISTs and their second primaries is not conspicuously attributed to the sharing of commonly seen oncogenic driver-gene mutations; this despite the nonrandom co-occurrence that has been described in institutional reports [4–6, 9, 20], multi-center analyses [7, 8], epidemiologic evidence and population-based studies [10].

Our study had several limitations. Firstly, our genetic study did not consider all kinds of associated second malignancies. Secondly, the genetic platform employed herein only surveyed the coding regions of 409 cancer-related genes; it is possible that shared mutations occurred at genomic regions not covered by our gene panel. Thirdly, the co-occurrence of GISTs and second malignancies may be attributable to certain single nucleotide polymorphisms that increase the risk of a patient developing GIST and other cancers, and which are better studied with a genome-wide association study. Lastly, epigenetic modifications may be involved in the development of dual cancers.

In conclusion, based on our extremely large single-institute series, 18.1% of patients with sporadic GISTs developed second primary cancers metachronously or synchronously, with GI and GU/GYN/breast cancers being the most frequently reported. The overall survival of patients with GISTs associated with second primaries was inferior to that of patients with GISTs alone, highlighting the clinical importance of this condition. Although population-based studies and case series worldwide, including ours, have unanimously indicated that the association is nonrandom, our targeted ultra-deep sequencing genomic screen revealed no clear driver-gene mutations that link sporadic GISTs and the most prevalent second primaries. Further studies are needed to explore other genetic alterations that may be responsible for this puzzling contradiction.

## MATERIALS AND METHODS

This retrospective study was approved by the Institutional Review Board at the Chang Gung Memorial Hospital (CGMH). A total of 749 patients with sporadic GISTs, who were surgically treated at the CGMH between 1999 and 2015, were enrolled into the study. The GIST diagnosis for all of these patients was histologically and immunohistochemically confirmed by CD117 and/or CD34 positivity. Of the 749 patients, those who had second primary malignancies with pathological confirmation were designated as our study cohort. These patients were categorized as follows: group 1, GIST before cancer(s); group 2, GIST after cancer(s); and group 3, synchronous occurrence of GIST and cancer(s), defined as an elapsed time interval between GIST and second malignancy emergence of 6 months or less. Notably, patients with a multi-neoplastic syndrome (for example Carney triad, type 1 neurofibromatosis, or familial GIST) were excluded. Clinicopathological parameters were recorded from the prospectively constructed electronic data bank [21, 22] as follows: age, gender, GIST location, tumor size, mitotic count, risk potential classification, immunohistochemical staining for KIT, CD34 and DOG1, and administration of TKIs such as imatinib methylate and/or sunitinib.

### Sample preparation, DNA sequencing and data processing

Surgically removed pre-treatment primary tumor tissue specimens were fixed in formalin and preserved in paraffin blocks. 10-μm-thick formalin-fixed paraffin-embedded (FFPE) sections were prepared, and genomic DNA was extracted from FFPE tumor samples using the QIAamp DNA FFPE Tissue Kit (Qiagen). DNA was quantified using the Quant-iT dsDNA High-Sensitivity Assay (Invitrogen). The integrity of genomic DNA was determined by capillary electrophoresis, using a Fragment Analyzer (Advanced Analytical Technologies, Inc. (AATI)). Eighty nanograms of genomic DNA was amplified using 4 pools of 15992 primer pairs (Ion AmpliSeq Comprehensive Cancer Panel, Life Technologies) to target the coding exon regions of 409 genes [23]. A detailed list of the genes studied is provided in Supplementary Table S1. Amplicons were ligated to barcoded adaptors using the Ion Amplicon Library Kit (Life Technologies). Barcoded libraries were subsequently conjugated to sequencing beads using emulsion PCR, and enriched using an Ion Chef system (Life Technologies) according to the Ion Torrent protocol (Life Technologies). The quality and quantity of the amplified library were determined using a Fragment Analyzer (AATI) and a Qubit fluorometer (Invitrogen). Sequencing was performed on an Ion Proton sequencer using the Ion PI chip (Life Technologies). Raw reads generated by the sequencer were mapped to the hg19 reference genome using the Ion Torrent Suite (v. 4.2). Coverage depth was calculated using the Torrent Coverage Analysis plug-in. Single nucleotide variants (SNVs) and short insertion/deletions were identified using the Torrent Variant Caller plug-in (version 4.2). Variant Effect Predictor (VEP, version 74) was used to annotate every variant using the following databases: COSMIC, v.70; dbSNP, build 138 and 1000 Genomes, phase. Variants with a coverage of less than 25, or a frequency of less than 5%, were filtered out.

### Statistical analysis

All data are presented as percentages or as means with standard deviation. Numerical data were tested for statistical significance using an independent two-sample test or ANOVA as appropriate. The Pearson chi-square test or Fisher's exact test were used to test for statistical significance when considering nominal variables. Survival rates were calculated, and plots constructed, using the Kaplan-Meier method, and groups were compared using a log-rank test. All statistical analyses were performed using the SPSS computer software package (Version 10.0, Chicago, IL, USA). A p-value <0.05 was considered statistically significant.

## SUPPLEMENTARY FIGURE AND TABLES






