# Supplementary material for: Targeted ultra-deep sequencing unveils a lack of driver-gene mutations linking non-hereditary gastrointestinal stromal tumors and highly prevalent second primary malignancies: random or nonrandom, that is the question
Source: Oncotarget. 2016 Oct 28;7(50):83270–7. doi: 10.18632/oncotarget.12452 (PMC5347768; doi:10.18632/oncotarget.12452)
Supplement: Supplementary file 1 [file oncotarget-07-83270-s001.pdf]

Targeted ultra-deep sequencing unveils a lack of driver-gene mutations linking non-hereditary gastrointestinal stromal tumors and highly prevalent second primary malignancies: random or nonrandom, that is the question

SUPPLEMENTARY FIGURE AND TABLES

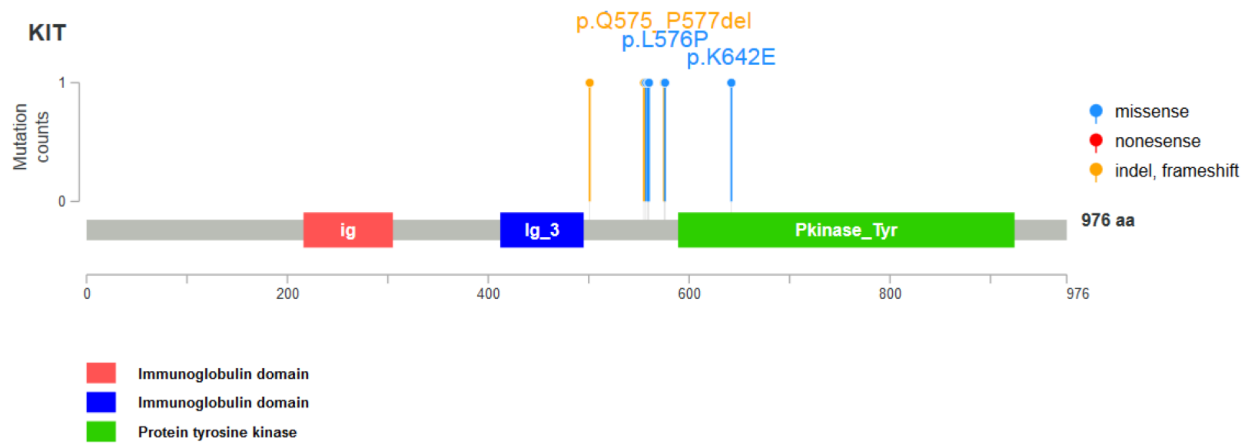

Supplementary Figure S1: Distribution of KIT genetic alterations detected in the 8 GISTs.

Supplementary Table S1: List of studied oncogenes and tumor suppressor genes.

See Supplementary File 1

Supplementary Table S2: Run Stats

Sample ID	Total read	On target reads	Mean depth	Uniformity
B00203	24741802	98.0%	1456	88.0%
B00204	26508176	98.4%	1603	92.0%
B00205	21022049	97.3%	1271	93.0%
B00206	18943520	98.3%	1164	91.8%
B00207	16647344	97.5%	1000	91.6%
B00208	16780993	98.0%	1015	91.5%
B00209	25786438	97.6%	1543	94.1%
B00314	16336394	97.9%	1000	93.6%
B00213	25534656	97.2%	1523	92.0%
B00214	17947783	98.0%	1091	93.0%
B00215	18391019	98.6%	1603	94.0%
B00216	28931635	98.7%	1788	88.4%
B00511	18380820	97.8%	1114	92.7%
B00315	16974791	98.2%	1018	89.7%
B00219	20539318	97.4%	1230	92.3%
B00220	25683358	98.0%	1490	87.0%
B00583	24347372	97.3%	1521	95.2%
B00585	24782566	97.8%	1563	95.0%

Supplementary Table S3: List of mutations.

See Supplementary File 2
