# Supplementary material for: Targeted ultra-deep sequencing unveils a lack of driver-gene mutations linking non-hereditary gastrointestinal stromal tumors and highly prevalent second primary malignancies: random or nonrandom, that is the question
Source: Oncotarget. 2016 Oct 28;7(50):83270–7. doi: 10.18632/oncotarget.12452 (PMC5347768; doi:10.18632/oncotarget.12452)
Supplement: Supplementary file 2 [file oncotarget-07-83270-s002.docx]

**Supplementary Table S1.** List of studied oncogenes and tumor suppressor genes

| *ABL1* | *BIRC5* | *CRKL* | *ETV4* | *HIF1A* | *KMT2C* | *MTOR* | *PAX7* | *RAD50* | *STK36* | *WHSC1* |
| --- | --- | --- | --- | --- | --- | --- | --- | --- | --- | --- |
| *ABL2* | *BLM* | *CRTC1* | *EXT1* | *HLF* | *KMT2D* | *MTR* | *PAX8* | *RAF1* | *SUFU* | *WRN* |
| *ACVR2A* | *BLNK* | *CSF1R* | *EXT2* | *HNF1A* | *KRAS* | *MTRR* | *PBRM1* | *RALGDS* | *SYK* | *WT1* |
| *ADAMTS20* | *BMPR1A* | *CSMD3* | *EZH2* | *HOOK3* | *LAMP1* | *MUC1* | *PBX1* | *RARA* | *SYNE1* | *XPA* |
| *AFF1* | *BRAF* | *CTNNA1* | *FANCA* | *HRAS* | *LCK* | *MUTYH* | *PDE4DIP* | *RB1* | *TAF1* | *XPC* |
| *AFF3* | *BRD3* | *CTNNB1* | *FANCC* | *HSP90AA1* | *LIFR* | *MYB* | *PDGFB* | *RECQL4* | *TAF1L* | *XPO1* |
| *AKAP9* | *BRIP1* | *CYLD* | *FANCD2* | *HSP90AB1* | *LPHN3* | *MYC* | *PDGFRA* | *REL* | *TAL1* | *XRCC2* |
| *AKT1* | *BTK* | *CYP2C19* | *FANCF* | *ICK* | *LPP* | *MYCL* | *PDGFRB* | *RET* | *TBX22* | *ZNF384* |
| *AKT2* | *BUB1B* | *CYP2D6* | *FANCG* | *IDH1* | *LRP1B* | *MYCN* | *PER1* | *RHOH* | *TCF12* | *ZNF521* |
| *AKT3* | *CARD11* | *DAXX* | *FAS* | *IDH2* | *LTF* | *MYD88* | *PGAP3* | *RNASEL* | *TCF3* |  |
| *ALK* | *CASC5* | *DCC* | *FBXW7* | *IGF1R* | *LTK* | *MYH11* | *PHOX2B* | *RNF2* | *TCF7L1* |  |
| *AMER1* | *CBL* | *DDB2* | *FGFR1* | *IGF2* | *MAF* | *MYH9* | *PIK3C2B* | *RNF213* | *TCF7L2* |  |
| *APC* | *CCND1* | *DDIT3* | *FGFR2* | *IGF2R* | *MAFB* | *NBN* | *PIK3CA* | *ROS1* | *TCL1A* |  |
| *AR* | *CCND2* | *DDR2* | *FGFR3* | *IKBKB* | *MAGEA1* | *NCOA1* | *PIK3CB* | *RPS6KA2* | *TET1* |  |
| *ARID1A* | *CCNE1* | *DEK* | *FGFR4* | *IKBKE* | *MAGI1* | *NCOA2* | *PIK3CD* | *RRM1* | *TET2* |  |
| *ARID2* | *CD79A* | *DICER1* | *FH* | *IKZF1* | *MALT1* | *NCOA4* | *PIK3CG* | *RUNX1* | *TFE3* |  |
| *ARNT* | *CD79B* | *DNMT3A* | *FLCN* | *IL2* | *MAML2* | *NF1* | *PIK3R1* | *RUNX1T1* | *TGFBR2* |  |
| *ASXL1* | *CDC73* | *DPYD* | *FLI1* | *IL21R* | *MAP2K1* | *NF2* | *PIK3R2* | *SAMD9* | *TGM7* |  |
| *ATF1* | *CDH1* | *DST* | *FLT1* | *IL6ST* | *MAP2K2* | *NFE2L2* | *PIM1* | *SBDS* | *THBS1* |  |
| *ATM* | *CDH11* | *EGFR* | *FLT3* | *IL7R* | *MAP2K4* | *NFKB1* | *PKHD1* | *SDHA* | *TIMP3* |  |
| *ATR* | *CDH2* | *EML4* | *FLT4* | *ING4* | *MAP3K7* | *NFKB2* | *PLAG1* | *SDHB* | *TLR4* |  |
| *ATRX* | *CDH20* | *EP300* | *FN1* | *IRF4* | *MAPK1* | *NIN* | *PLCG1* | *SDHC* | *TLX1* |  |
| *AURKA* | *CDH5* | *EP400* | *FOXL2* | *IRS2* | *MAPK8* | *NKX2-1* | *PLEKHG5* | *SDHD* | *TNFAIP3* |  |
| *AURKB* | *CDK12* | *EPHA3* | *FOXO1* | *ITGA10* | *MARK1* | *NLRP1* | *PML* | *SEPT9* | *TNFRSF14* |  |
| *AURKC* | *CDK4* | *EPHA7* | *FOXO3* | *ITGA9* | *MARK4* | *NOTCH1* | *PMS1* | *SETD2* | *TNK2* |  |
| *AXL* | *CDK6* | *EPHB1* | *FOXP1* | *ITGB2* | *MBD1* | *NOTCH2* | *PMS2* | *SF3B1* | *TOP1* |  |
| *BAI3* | *CDK8* | *EPHB4* | *FOXP4* | *ITGB3* | *MCL1* | *NOTCH4* | *POT1* | *SGK1* | *TP53* |  |
| *BAP1* | *CDKN2A* | *EPHB6* | *FZR1* | *JAK1* | *MDM2* | *NPM1* | *POU5F1* | *SH2D1A* | *TPR* |  |
| *BCL10* | *CDKN2B* | *ERBB2* | *G6PD* | *JAK2* | *MDM4* | *NRAS* | *PPARG* | *SMAD2* | *TRIM24* |  |
| *BCL11A* | *CDKN2C* | *ERBB3* | *GATA1* | *JAK3* | *MEN1* | *NSD1* | *PPP2R1A* | *SMAD4* | *TRIM33* |  |
| *BCL11B* | *CEBPA* | *ERBB4* | *GATA2* | *JUN* | *MET* | *NTRK1* | *PRDM1* | *SMARCA4* | *TRIP11* |  |
| *BCL2* | *CHEK1* | *ERCC1* | *GATA3* | *KAT6A* | *MITF* | *NTRK3* | *PRKAR1A* | *SMARCB1* | *TRRAP* |  |
| *BCL2L1* | *CHEK2* | *ERCC2* | *GDNF* | *KAT6B* | *MLH1* | *NUMA1* | *PRKDC* | *SMO* | *TSC1* |  |
| *BCL2L2* | *CIC* | *ERCC3* | *GNA11* | *KDM5C* | *MLLT10* | *NUP214* | *PSIP1* | *SMUG1* | *TSC2* |  |
| *BCL3* | *CKS1B* | *ERCC4* | *GNAQ* | *KDM6A* | *MMP2* | *NUP98* | *PTCH1* | *SOCS1* | *TSHR* |  |
| *BCL6* | *CMPK1* | *ERCC5* | *GNAS* | *KDR* | *MN1* | *PAK3* | *PTEN* | *SOX11* | *UBR5* |  |
| *BCL9* | *COL1A1* | *ERG* | *GPR124* | *KEAP1* | *MPL* | *PALB2* | *PTGS2* | *SOX2* | *UGT1A1* |  |
| *BCR* | *CRBN* | *ESR1* | *GRM8* | *KIT* | *MRE11A* | *PARP1* | *PTPN11* | *SRC* | *USP9X* |  |
| *BIRC2* | *CREB1* | *ETS1* | *GUCY1A2* | *KLF6* | *MSH2* | *PAX3* | *PTPRD* | *SSX1* | *VHL* |  |
| *BIRC3* | *CREBBP* | *ETV1* | *HCAR1* | *KMT2A* | *MSH6* | *PAX5* | *PTPRT* | *STK11* | *WAS* |  |
