# Supplementary material for: Targeted ultra-deep sequencing unveils a lack of driver-gene mutations linking non-hereditary gastrointestinal stromal tumors and highly prevalent second primary malignancies: random or nonrandom, that is the question
Source: Oncotarget. 2016 Oct 28;7(50):83270–7. doi: 10.18632/oncotarget.12452 (PMC5347768; doi:10.18632/oncotarget.12452)
Supplement: Supplementary file 3 [file oncotarget-07-83270-s003.docx]

**Supplementary Table S3. List of mutations**
